# Proficiency testing within Eurotransplant

**DOI:** 10.3389/fgene.2024.1451748

**Published:** 2024-09-23

**Authors:** Yvonne M. Zoet, Sebastiaan Heidt, Marissa J. H. van der Linden-van Oevelen, Geert W. Haasnoot, Frans H. J. Claas

**Affiliations:** ^1^ Immunology-ETRL, Leiden University Medical Center (LUMC), Leiden, Netherlands; ^2^ Eurotransplant, Leiden, Netherlands

**Keywords:** proficiency test, solid organ transplantation, histocompatibility testing, Eurotransplant, patient-based cases

## Abstract

Eurotransplant is responsible for the international allocation of organs between eight countries in Europe. All HLA laboratories affiliated to Eurotransplant must be EFI or ASHI-accredited and must participate in the Eurotransplant external proficiency testing (EPT) program, organized by the Eurotransplant Reference Laboratory (ETRL). EPT within Eurotransplant has a long tradition, starting in 1978. The current EPT program consists of the following schemes: HLA typing including serology, CDC crossmatching, HLA-specific antibody detection, and identification. Participants enter the results of laboratory tests using a web-based application. Assessed results are visible on the website. An additional component called “patient-based cases” runs since 2016. Results are summarized and published on the EPT website. Furthermore, these results are discussed during the annual extramural tissue typers meeting, which is organized by the ETRL. Thanks to this EPT program, the performance of all HLA laboratories affiliated to Eurotransplant can be monitored and corrected, if necessary. Because all affiliated laboratories are assessed in the same EPT program, where these laboratories show to be consistent in most of their results, Eurotransplant EPT has proven to be an efficient tool to create a more uniform level of quality of histocompatibility testing within Eurotransplant.

## Introduction

Eurotransplant is an international organ exchange organization founded in 1967 by prof. Dr. Jon van Rood (van Rood, 1967). Currently, Eurotransplant consists of eight member states: Austria, Belgium, Croatia, Germany, Hungary, Luxemburg, Slovenia, and the Netherlands. Histocompatibility plays an important role in the allocation of most types of organs, and in total, 44 tissue typing laboratories affiliated to the transplant centers participate in Eurotransplant. In order to guarantee a uniform level of histocompatibility testing, the Eurotransplant external proficiency testing started in 1978 (Schreuder et al., 1986), with an exchange of cell material for serological HLA typing. Later, EPT on HLA-specific antibody detection and identification and EPT on crossmatching were introduced (Doxiadis et al., 2000).

Nowadays, three different schemes are running: the EPT scheme for HLA typing and crossmatching, with four dispatches per year; and the EPT scheme for HLA-specific antibody detection and identification, for which 12 different sera are shipped. The latest scheme is the patient-based case scheme, which started with a pilot in 2015 and was officially introduced in 2016. These EPT schemes cover the most frequently used techniques in laboratory testing for solid organ transplantation (Bontadini, 2012).

All schemes follow the recommendations of the European Federation for Immunogenetics (EFI), as documented in both the EFI standards and the EFI standards for EPT providers (https://efi-web.org/). All laboratories affiliated to Eurotransplant and most of the other participating laboratories are EFI-accredited, for which EPT plays a significant role (Harmer, Mascaretti, and Petershofen, 2018). The Eurotransplant Tissue Typing Advisory Committee (TTAC) has an advisory role for all histocompatibility-related activities within Eurotransplant and has to approve major changes in the EPT scheme.

Additionally, all EPT schemes organized by the ETRL follow the policies of Eurotransplant. The schemes are unique because they give the possibility to compare the laboratories affiliated to Eurotransplant. However, the Eurotransplant EPT is also open for Histocompatibility and Immunogenetics (H&I) laboratories from countries outside of Eurotransplant. In total, 44 Eurotransplant-affiliated laboratories and 36 laboratories from outside the Eurotransplant area are participating in one or more of the schemes organized. All schemes are mandatory for Eurotransplant-affiliated laboratories, while the remaining laboratories can choose to participate in one or more of the schemes. Patient-based cases are also open to participants outside of Eurotransplant, taking part in either the HLA typing and crossmatching scheme and/or in the HLA-specific antibody detection and identification scheme. The results from all EPT schemes are published on the EPT website. Furthermore, during the yearly Eurotransplant extramural tissue typers meeting, the results from all EPT schemes are discussed with the representatives of the different tissue typing laboratories.

## Materials and methods

### EPT on HLA typing

Every year four sets of three blood samples are shipped to the participants. Characteristics are depicted in Table 1. HLA typing results are to be entered in the format of Eurotransplant Match Determinants, based on the 2008 HLA dictionary (Holdsworth et al., 2009), as well as on serotypes as published by Osoegawa et al. (2022).

**TABLE 1:** Overview of EPT schemes and their characteristics.

	Scheme 1		Scheme 2		Scheme 3
	HLA typing	Crossmatching	HLA-specific antibody detection	HLA-specific antibody identification	Patient-based cases
Dispatches per year	4	4	1	1	3
Numbers and types of samples per dispatch	3 tubes of blood	3 tubes of blood 3 serum samples	12 vials of serum in one shipment	12 vials of serum in one shipment	n.a
Period until deadline	2 weeks	2 weeks	4–5 months	4–5 months	2 weeks
Entrance of results	Manually on the website	Manually on the website	Manually on the website	Manually on the website	By e-mail
Entrance of results	Eurotransplant match determinants	Positive/negative	Positive/negative	Specificities for HLA-A, -B, -C, -DR, -DQ, and -DP	Answer and motivation related to the case
Time for the assessment of the results	2 weeks; reports are published on the EPT website	2 weeks; reports are published on the EPT website	1 month; reports are published on the EPT website	1 month; reports are published on the EPT website	2 months; the summary is published on the EPT website
Assessment method	Based on 75% consensus	Based on 75% consensus	Detection 75% consensus	Identification CDC: 75% consensus; bead-based methods: 95% consensus	Not assessed; a summary with all the results is published
Maximum yearly discrepancy rate	10%	15%	20%	25%	n.a
Methods assessed	CDC and low-resolution HLA typing	CDC	Bead-based methods, CDC	CDC, single antigen bead assays and complement-dependent SA bead assays	n.a
Number of participants in 2023	68	66	76	76	52

Results can be entered manually and authorized on the ETRL-EPT website. The deadline for the submission of the results is 2 weeks from the day of shipment. After the deadline, results are assessed on the basis of consensus, as described in the EFI standards for providers. In case no consensus is reached, reference typing is done by the ETRL.

### EPT on CDC crossmatching

For crossmatching, four shipments consisting of three blood samples and three serum samples per year are sent to the participating laboratories. For an overview, see Table 1. Crossmatching on either T cells or unseparated cells is mandatory for all Eurotransplant-affiliated laboratories. All participating laboratories can perform B-cell crossmatches as well. Results are entered on the ETRL-EPT website. The deadline is 2 weeks from the day of shipment. Crossmatch results are assessed on the basis of a 75% consensus.

### EPT on HLA-specific antibody detection

In total, 12 serum samples are sent yearly for the EPT on HLA-specific antibody detection. The characteristics are shown in Table 1. Eurotransplant-affiliated participants must use a CDC-based technique. Additionally, it is possible to use Luminex bead-based, flow cytometric-based, and ELISA-based techniques. Results are to be entered as positive (HLA-specific antibodies present) or negative (absence of HLA-specific antibodies) on the ETRL-EPT website. The period until the deadline is 4 to 5 months from the day of shipment.

### EPT on HLA-specific antibody identification

For the EPT on HLA-specific antibody identification, the same 12 sera are used as shipped for the EPT on HLA-specific antibody detection. More information is given in Table 1. For HLA-specific antibody identification, Eurotransplant-affiliated laboratories must use CDC as a method. Last year, 11 laboratories from outside Eurotransplant used CDC as a technique. Next to this, Luminex single-antigen bead (SAB) assays and Luminex SAB complement binding assays are assessed separately. An assessment is done on the basis of consensus. The ETRL uses 75% consensus for CDC and 95% consensus for SAB assays. Results are entered on the ETRL-EPT website. The period until the deadline is 4 to 5 months from the day of shipment.

### Patient-based cases EPT

The Eurotransplant patient-based cases EPT started with a pilot in 2015. This was done because the need was felt to not only produce laboratory results of crossmatching, HLA typing, and HLA-specific antibody detection and identification but to also have an EPT that asks for the interpretation of these results in order to assess the (level of) histocompatibility between a patient and a potential donor.

Two pilot cases were sent out. The first pilot case consisted of patient and donor information and laboratory data, such as HLA typing, immunizing events, HLA-specific antibody identification data, and crossmatch results. Participants could interpret the complete dataset and judge whether the donor offer was suitable for the patient and motivate their answer.

The second pilot case was a combination of the regular EPT on HLA typing and crossmatching in combination with the question to select the best suitable patient for a donor. Both crossmatches and donor HLA typing were performed by the participating centers. Other data like recipient HLA types were given.

After this pilot, it was decided to continue with the exercises solely based on the results that were already available, as in the first pilot case. Combined exercises (second pilot case) were stopped because with only few available data (HLA typing and crossmatch results), it was difficult to create realistic scenarios.

In 2016, the patient-based case EPT was formalized. Since then, this EPT is mandatory for all Eurotransplant-affiliated laboratories, and a yearly certificate of participation is issued. The EPT consists of three patient cases each year. The focus is on transplantation with kidneys from deceased donors as the Eurotransplant-affiliated laboratories have testing for this goal as one of their main tasks.

Over time, other scenarios were added, e.g., to give attention to specific Eurotransplant policies, upcoming changes in Eurotransplant or Eurotransplant programs, such as the AM program (Heidt et al., 2021), and the recently introduced virtual donor crossmatch (Claas and Heidt, 2020).

### Certificates

Certificates are issued by the ETRL based on the performance in the EPT for both the HLA typing and crossmatching scheme and for the HLA-specific antibody detection and identification scheme. The following results on the certificate are possible: fulfilled/not fulfilled/participated. The certificate for (not) fulfilling the requirements can only be awarded if a minimum number of 10 participants report results in the respective category. For certificates of successful performance (fulfilled), the participant needs to meet the criteria, as described in the latest version of the Eurotransplant Manual, the latest version of the EFI standards, and the latest version of the EFI standards for providers. When for a given technique less than 10 participants join the EPT, a participation certificate will be issued. For the patient-based cases EPT, a certificate of participation is issued.

## Results and discussion

The present results have been partly published in the Eurotransplant Annual Report 2023 (www.Eurotransplant.org).

In general, no significant differences between the eight Eurotransplant member states are observed, and there are no large differences in the results between Eurotransplant-affiliated laboratories and all other laboratories. Overall, the participants in the Eurotransplant EPT have satisfying results, with occasional exceptions.

### EPT on HLA typing

**FIGURE 1:**
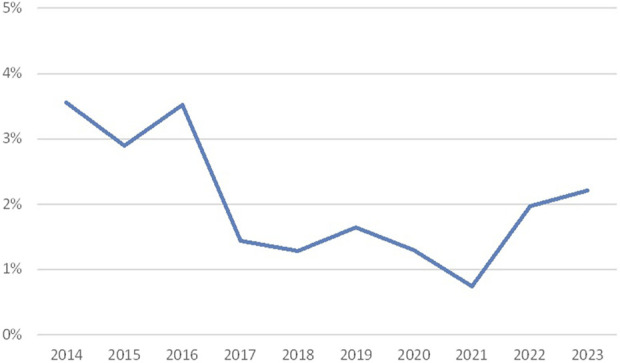
Discrepancy rates in HLA typing (percentage discrepancies over the years 2014–2023; source Eurotransplant).

The results of the HLA typing scheme are shown in Figure 1. Since the observation that DNA-based HLA typing is more reliable than serological typing (Opelz et al., 1991), molecular typing has been introduced within Eurotransplant, leading to a diminished discrepancy rate (3.6% in the period 2014–2023). The observed discrepancies are most often clerical errors, such as errors occurring during the manual entrance of the results. Other more often observed errors are mix-up of samples and entering a broad when a split can be detected (e.g., Cw3 instead of Cw10 (Cw3).

The decrease in discrepancy rates until 2021 can partly be explained by the fact that a Eurotransplant policy was introduced to only assign Bw4 together with B-locus antigens. During a few years, the participants have gradually incorporated this. The increase in the following years is probably related to the introduction of the virtual crossmatch. Since that moment DQA1, DPB1, and DPA1 results had to be reported as well.

With the introduction of the virtual crossmatch in Eurotransplant, donor HLA types can be electronically transmitted. A discussion has started whether this may be feasible for the EPT on HLA typing in the future. A possible advantage could be that this way clerical errors will be avoided.

### EPT on CDC crossmatching

**FIGURE 2:**
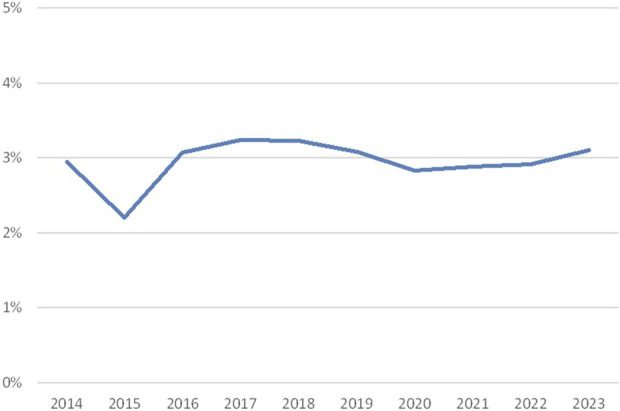
Discrepancy rates crossmatching (percentage discrepancies over the years 2014–2023; source Eurotransplant).

Overall, approximately 3% discrepancy rates were observed in the period 2014–2023 (Figure 2), with an exception in 2015 (2.2%). There is no clear explanation, such as lower consensus rates, for this lower percentage.

Discrepancies in crossmatching may have various causes. Since for crossmatching fresh blood is shipped, the duration of the shipment can influence the quality of the target cells. Moreover, crossmatching is done by CDC-based methods, which may vary with respect to the technique itself and the interpretation of the results, depending on the technician and her or his experience. In addition, a difference is seen between the cell types used for crossmatching. The comparison of B-cell crossmatches to T-cell crossmatches and unseparated cell crossmatches is challenging, leading either to more discrepancies or to results that are not in consensus. To this end, B-cell crossmatches are analyzed separately.

Consensus rates (Supplementary Figure S1) were around 90% in the period 2014–2023, with higher consensus rates for unseparated cell and T-cell crossmatching and lower (but usually not below 75%) for B-cell crossmatching.

### EPT on HLA-specific antibody detection

**FIGURE 3:**
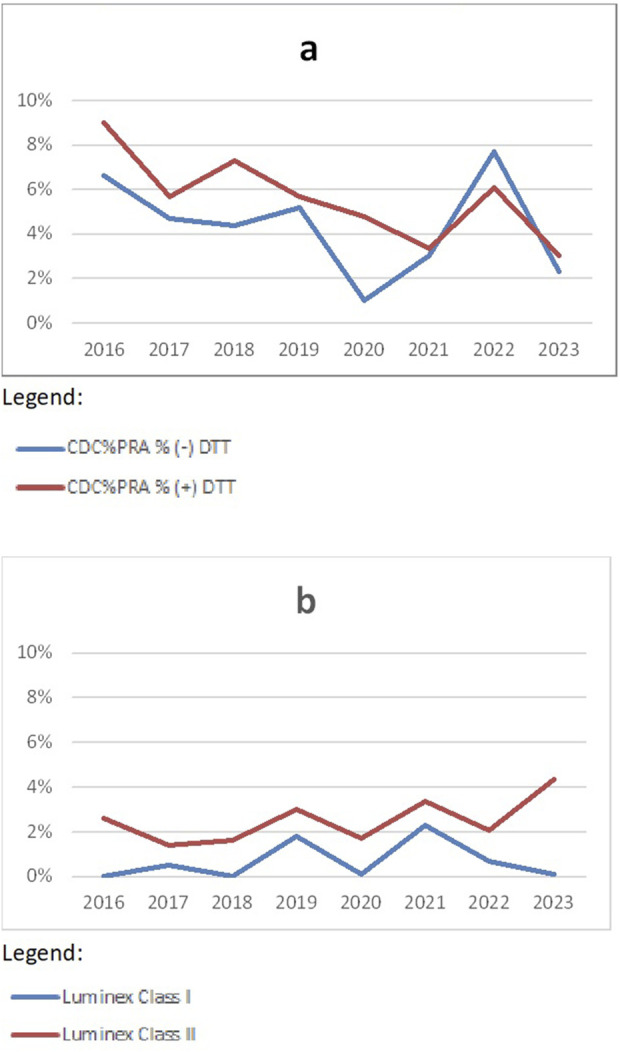
Discrepancy rates in HLA-specific antibody detection CDC **(A)** and methods **(B)** over the years 2016–2023 (source Eurotransplant).

When looking at the detection of HLA-specific antibodies (Figure 3), it is obvious that more discrepancies are found in CDC-based methods (Figure 3A, discrepancy rates around 5%) compared to Luminex-based methods (Figure 3B, discrepancy rates around 1.5%). Currently, there are two vendors of Luminex-based antibody screening kits, which differ in performance and sensitivity. When discrepancies are seen in Luminex-based methods, this is often caused by one kit being more sensitive than the other kit. Since separated analysis for CDC and SAB techniques was introduced in 2016, only results from 2016–2023 are shown.

### EPT on HLA-specific antibody identification

The CDC-based HLA-specific antibody identification results in more false-negative and false-positive results relative to the total amount of consensus specificities found compared to SAB techniques as long as this is analyzed (over 10 years). The number of consensus specificities in CDC-based HLA-specific antibody identification was between 11 and 26 (average 17 specificities) in the period 2014–2023. In addition, more laboratories with less satisfactory results in CDC-based HLA-specific antibody identification are seen (Table 2). This can be explained by variation in the cell panel size and used HLA types in cell panels. Around 50% of the ET-affiliated laboratories use an in-house panel (information from an ETRL inventory in 2023), and the other half of ET-affiliated laboratories use CDC tests from different companies.

**TABLE 2:** Percentages of participants with unsatisfactory results in the period 2019–2023.

	HLA typing (%)	Crossmatching (%)	HLA-specific antibody detection (%)	HLA-specific antibody identification CDC (%)	HLA-specific antibody identification SPA SA (%)
% Participants with unsatisfactory results	3.2	1.2	2.4	9.0	0.3

**FIGURE 4:**
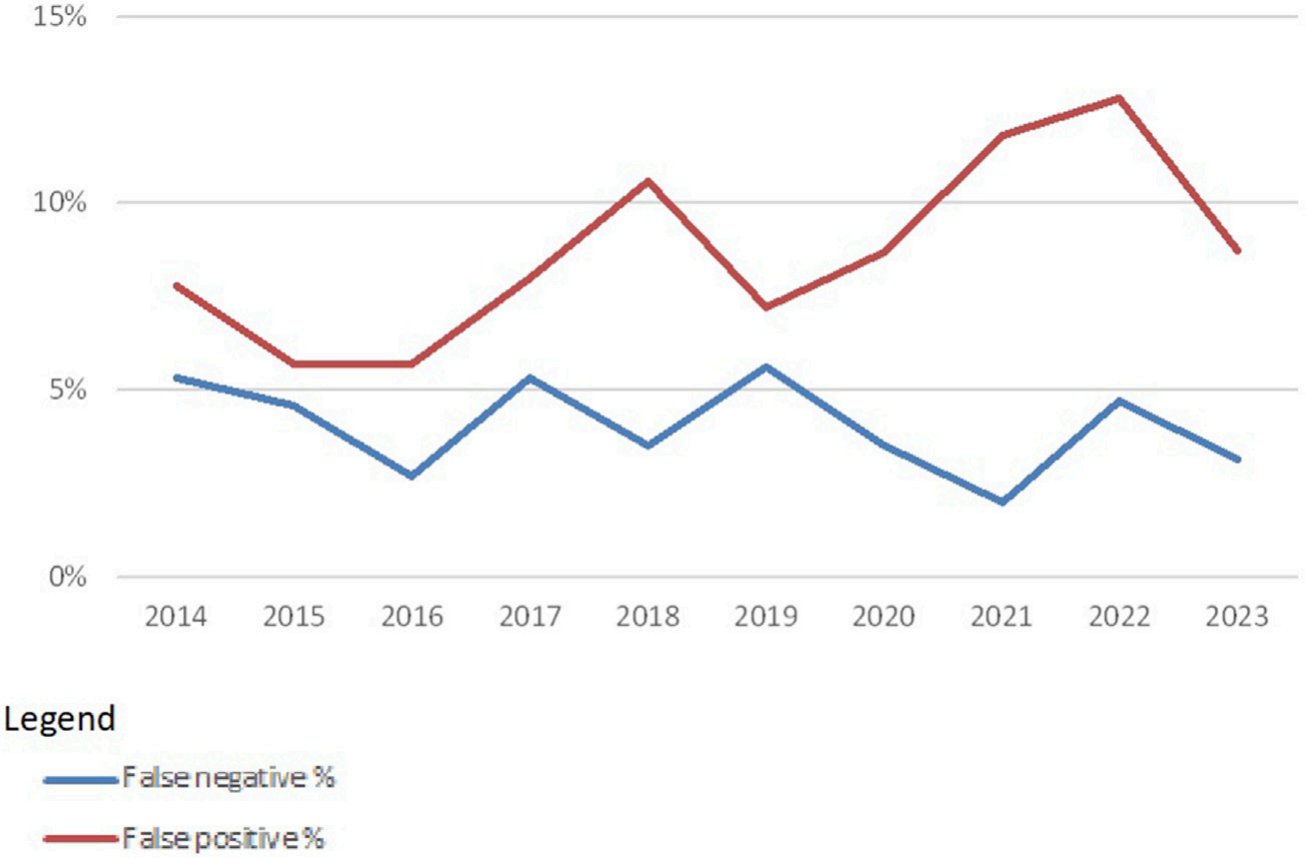
False-negative and false-positive rates in HLA-specific antibody identification with CDC-based methods over the years 2014–2023 (source Eurotransplant).

Overall, this leads to false-negative rates of around 4% and false-positive rates of around 10% (Figure 4). False positives are extra specificities found by the participating laboratories. The higher (10%–12%) percentages seen here are mainly due to a few participants reporting several specificities detected via SAB techniques in the CDC proficiency tests. False negatives are missed specificities. This percentage (around 4%) is much lower, which may indicate that most laboratories are indeed capable of detecting relevant specificities with CDC, which shows that this technique is valuable for the future.

The false-negative and false-positive rates in SAB techniques are much lower mainly because more consensus specificities are found in the period 2014–2023. The average number of consensus specificities for HLA Class I is 279 and the average number for consensus specificities for HLA Class II is 55. In 2023, also DQA1, DPA1, and DPB1 specificities could be entered, resulting in 102 consensus specificities for Class II. A second reason why these false-negative and false-positive rates are lower is that every laboratory is using one of the two commercially available kits, whereas CDC cell panels vary from laboratory to laboratory.

**FIGURE 5:**
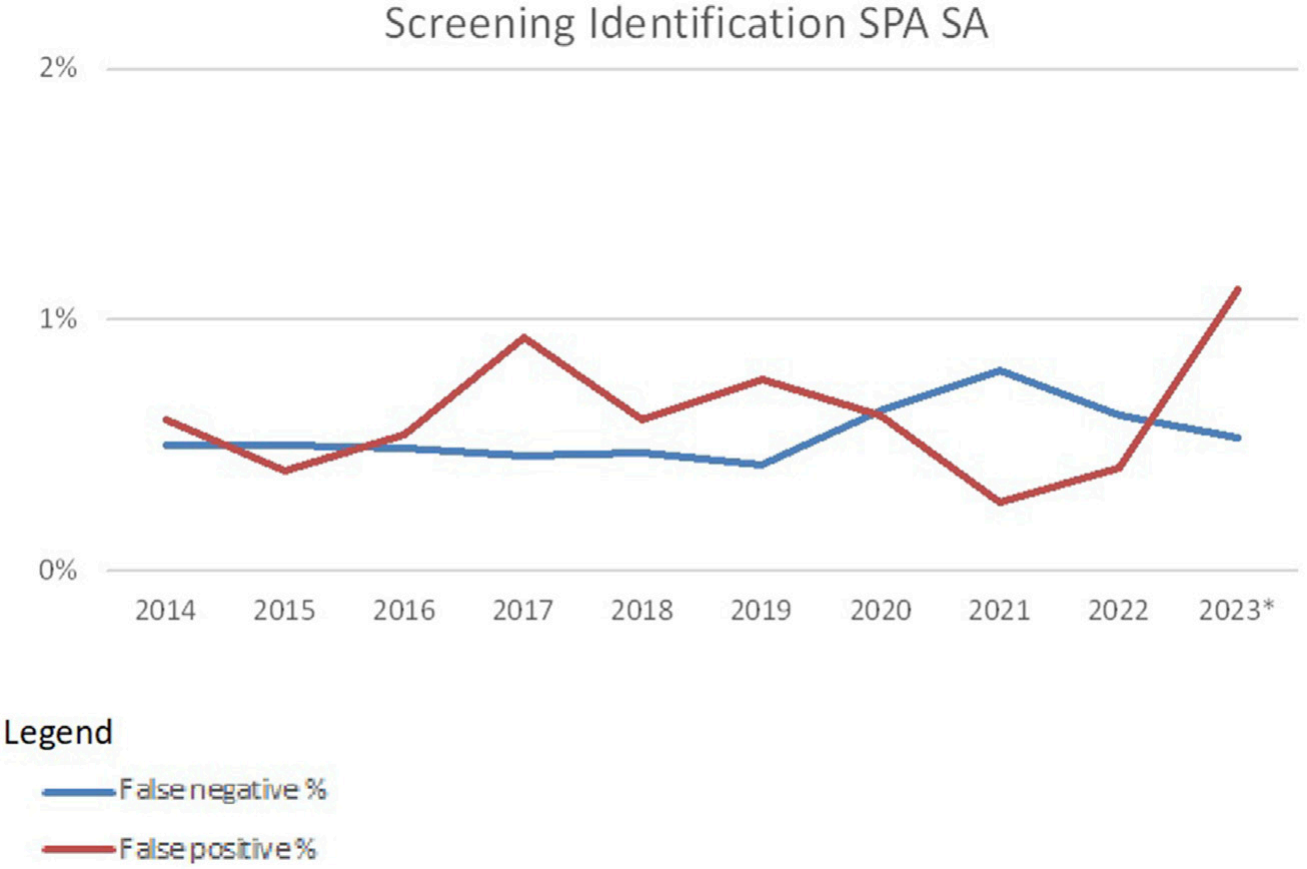
False-negative and false-positive rates in HLA-specific antibody identification with SAB-based methods over the years 2014–2023 (source Eurotransplant). *As of 2023 DQA, DPA and DPB specificities were included.

False-negative rates and false-positive rates for SAB techniques are usually around 0.6% (Figure 5). The introduction of additional specificities (DQA1, DPA1, and DPB1) led to a higher false-positive rate in 2023. False positives for SAB techniques could be due to low or very low cut-off values used in some of the laboratories. In addition, for SAB tests, the market is dominated by only two vendors. When using identical cut-off values in tests from both vendors, misinterpretation may be a consequence (Karahan et al., 2023).

**FIGURE 6:**
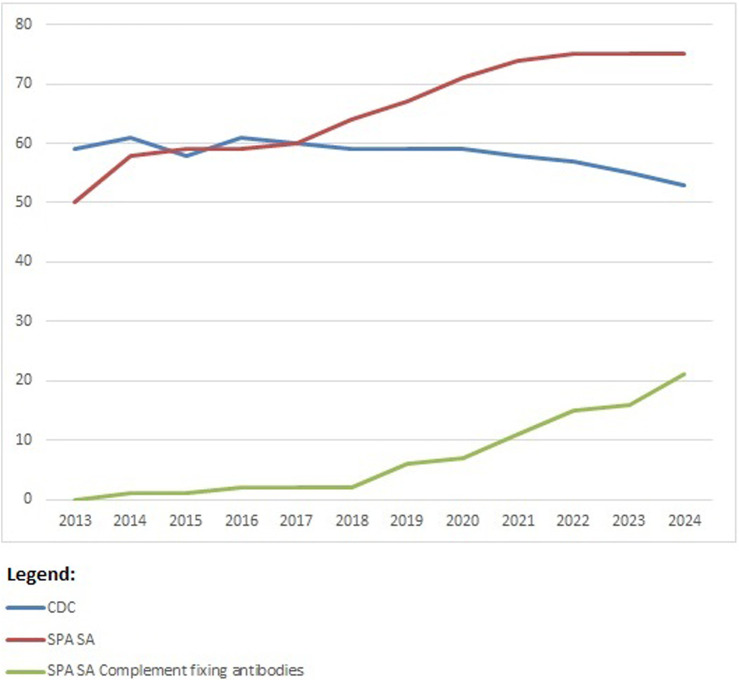
Participation per technique for HLA-specific antibody identification over the years 2013–2023; number of participants on the vertical axis.

From 2013 until present, there is a trend toward using SAB methods by almost all participants (Figure 6). Within Eurotransplant, CDC is used together with SAB methods to have more complete information about the impact of the antibodies detected and possibly exclude denatured antibodies found in SAB methods. Therefore, CDC is still a mandatory technique for the Eurotransplant-affiliated laboratories. Outside Eurotransplant, 11 participants were using CDC as one of the techniques used in HLA antibody identification in 2023. The complement-fixing SAB techniques are used more and more, especially outside Eurotransplant (11/16 participants are not affiliated to Eurotransplant).

A future prospect may be the combination of CDC and SAB techniques in order to define acceptable and/or unacceptable antigens.

### Patient-based case EPT

The results of the patient-based cases are reported to the participants by means of a summary, which is published on the EPT website. The information in the summaries consists of a short description of the case itself. Next to this, a graph is made to show an overview of the final answer (e.g., transplanting yes, no, or may be). Furthermore, for all the categories of final answers, observations and motivations are sorted, counted, and listed. Then, a list of missing information (according to the participants) and a list of recommendations are given. Finally, an ETRL comment is given, e.g., with the outcome of a transplantation or information about Eurotransplant policies.

All patient-based cases are presented and discussed during the Eurotransplant extramural tissue typers meeting. The same holds true for all other EPT results. This meeting is meant for all staff in the Eurotransplant-affiliated laboratories and is organized yearly by the ETRL together with one of the affiliated laboratories.

The presentation and discussion of the patient-based cases results reveal differences in policies between transplantation centers and affiliated tissue typing laboratories and provide insights why certain decisions are taken. These discussions are very important and create a more solid basis for a proper interpretation of the laboratory results.

Whenever there is a new policy in Eurotransplant, the patient-based case EPT is used to practice with this upcoming policy and is used to familiarize the affiliated transplant centers with the policies. Another practical use of these cases is to give the participants the possibility to practice with ETRL/Eurotransplant tools such as the calculators for donor frequency and virtual panel reactive antibodies (vPRAs).

The results for the patient-based case EPT are not assessed, like in some patient-based cases EPT in clinical chemistry laboratories (Sciacovelli et al., 2003). The main reasons why the cases are not assessed are as follows:

• Centers within Eurotransplant are allowed to have their own policy regarding the acceptance of donors as long as the rules in the Eurotransplant manual are followed. This means that there can be more than one correct answer.
• An assessment of the cases may prevent an open discussion on the pros and cons of different decisions.
• An assessment of such cases is complicated and would require a team judging the cases before and determining the best assessment criteria, which is prone to being subjective.

### Certificates

In general, most participants have satisfying results for different ETRL-EPT schemes, which leads to a certificate stating “fulfilled.” In the years 2019–2023, 3.2% of the participants had unsatisfactory results for typing, and 1.2% of the participants failed to meet the criteria for crossmatching. In total, 2.4% of the participants had poor results for HLA-specific antibody screening detection, and for HLA-specific antibody identification, the percentages for unsatisfactory results were 9.0% and 0.3%, respectively (Table 2). These percentages are quite stable over this 5-year period. Some fluctuations in CDC HLA-specific antibody identification were observed. This can be explained by the variation in HLA-specific antibody specificities of the serum samples shipped. It is known that not all CDC cell panels allow for the detection of all HLA-specific antibody specificities. In particular, HLA-C specificities are not always detected by all participants, most likely due to the lower expression of HLA-C compared to HLA-A and HLA-B on the cell surface.

Whenever participants have unsatisfactory results and receive a certificate stating “not fulfilled,” they have the option to join the extra EPT, which is organized by the ETRL. This extra EPT gives the opportunity to show that unsatisfactory scoring was of a temporary nature. Additionally, this extra EPT serves those participants who could not test all samples of the regular shipment(s) because of problems in the transportation of the material.

## Conclusion

The Eurotransplant EPT program has shown its benefit with respect to the performance of the individual laboratories affiliated to Eurotransplant and the interpretation of the results. Laboratory results incidentally are below a satisfactory level but usually return to a satisfactory level in the next year or in the extra EPT exercise at the end of the year organized by the ETRL. The discussion of both the EPT results and the patient cases during the yearly extramural meeting makes all participants aware of the fact that there are still differences in policies with respect to decision-making in different transplant centers. The discussion of these differences is very useful for the critical evaluation of existing policies in the local centers, but it also leads to novel insights, especially with respect to the interpretation of the clinical relevance of HLA antibody reactivities. In particular, the translation of an HLA-specific antibody specificity detected in Luminex SAB assays into an acceptable or non-acceptable HLA antigen will be an important part of the discussion in the coming years, considering the recent introduction of virtual crossmatching within Eurotransplant.
