# Supplementary figures and images for: Proficiency testing within Eurotransplant
Source: Front Genet. 2024 Sep 23;15:1451748. doi: 10.3389/fgene.2024.1451748 (PMC11456461; doi:10.3389/fgene.2024.1451748)

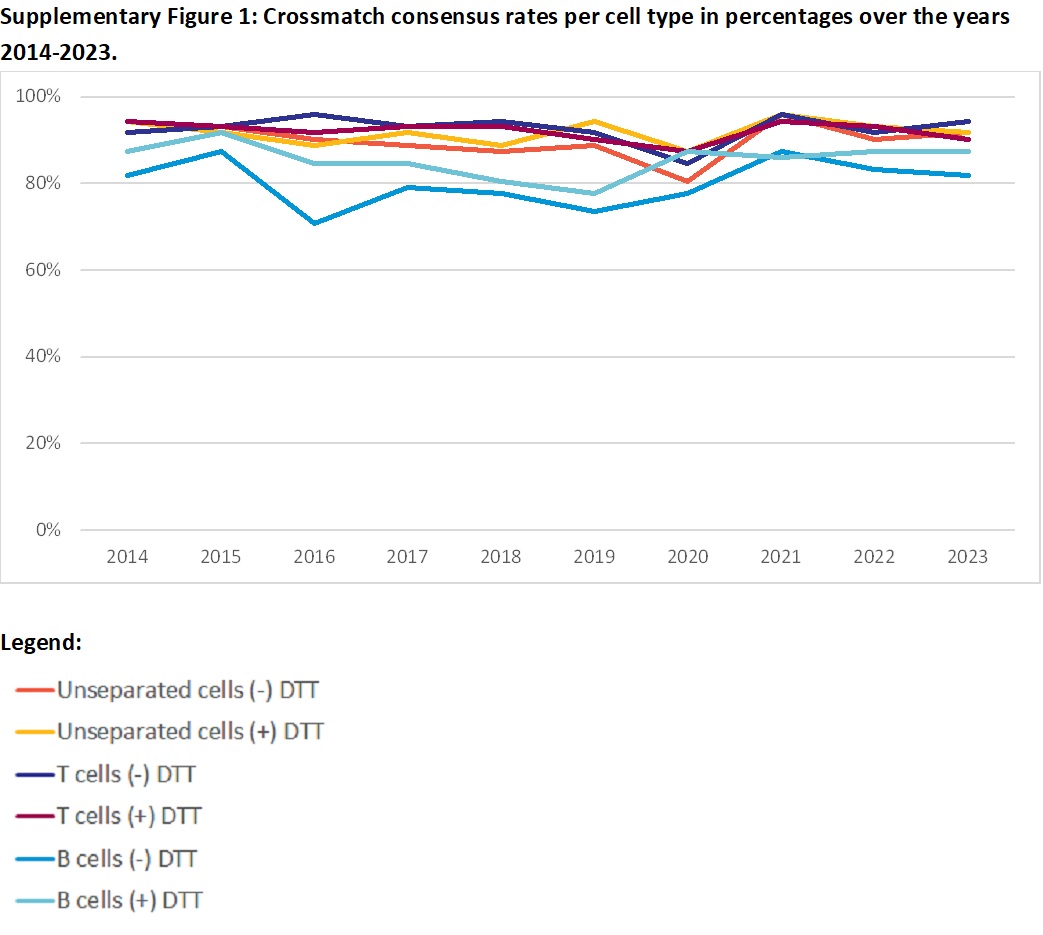

Supplement: Supplementary file 1 [file Image1.JPEG]
